# Applications of MALDI-TOF Mass Spectrometry to the Identification of Parasites and Arthropod Vectors of Human Diseases

**DOI:** 10.3390/microorganisms10112300

**Published:** 2022-11-20

**Authors:** Fernando Sánchez-Juanes, Noelia Calvo Sánchez, Moncef Belhassen García, Carmen Vieira Lista, Raul Manzano Román, Rufino Álamo Sanz, Antonio Muro Álvarez, Juan Luis Muñoz Bellido

**Affiliations:** 1 Department of Biochemistry and Molecular Biology, University of Salamanca, 37007 Salamanca, Spain; 2 Department of Microbiology, Hospital Universitario de Salamanca, 37007 Salamanca, Spain; 3 Department of Medicine-Infectious Diseases, Hospital Universitario de Salamanca, 37007 Salamanca, Spain; 4 Centro de Investigación en Enfermedades Tropicales de la Universidad de Salamanca (CIETUS), Universidad de Salamanca, 37008 Salamanca, Spain; 5 Instituto de Investigación Biomédica de Salamanca (IBSAL), Hospital Universitario de Salamanca, Universidad de Salamanca, CSIC, 37007 Salamanca, Spain; 6 Public Health Information Service, Consejería de Sanidad, Junta de Castilla y León, 47007 Valladolid, Spain

**Keywords:** MALDI-TOF MS, parasites, arthropods

## Abstract

Arthropod vectors and parasites are identified morphologically or, more recently, by molecular methods. Both methods are time consuming and require expertise and, in the case of molecular methods, specific devices. Matrix-assisted laser desorption ionization time-of-flight mass spectrometry (MALDI-TOF MS) identification of bacteria has meant a major change in clinical microbiology laboratories because of its simplicity, speed and specificity, and its capacity to identify microorganisms, in some cases, directly from the sample (urine cultures, blood cultures). Recently, MALDI-TOF MS has been shown as useful for the identification of some parasites. On the other hand, the identification of vector arthropods and the control of their populations is essential for the control of diseases transmitted by arthropods, and in this aspect, it is crucial to have fast, simple and reliable methods for their identification. Ticks are blood-sucking arthropods with a worldwide distribution, that behave as efficient vectors of a wide group of human and animal pathogens, including bacteria, protozoa, viruses, and even helminths. They are capable of parasitizing numerous species of mammals, birds and reptiles. They constitute the second group of vectors of human diseases, after mosquitoes. MALDI-TOF MS has been shown as useful for the identification of different tick species, such as *Ixodes*, *Rhipicephalus* and *Amblyomma*. Some studies even suggest the possibility of being able to determine, through MALDI-TOF MS, if the arthropod is a carrier of certain microorganisms. Regarding mosquitoes, the main group of vector arthropods, the possibility of using MALDI-TOF MS for the identification of different species of *Aedes* and *Anopheles* has also been demonstrated. In this review, we address the possibilities of this technology for the identification of parasites and arthropod vectors, its characteristics, advantages and possible limitations.

## 1. Introduction

MALDI-TOF mass spectrometry (MS) has established itself in recent years as an extraordinarily useful resource for bacterial identification, due to its reliability and speed [1,2,3]. MALDI-TOF MS uses protein profiles that are characteristic of each species, for bacterial identification. The oldest studies measured proteins with lower molecular weights [4], while the most modern systems use a range of 2000–20,000 Da. This range of molecular weight predominantly represents ribosomal proteins obtained from whole bacterial extracts [5]. These proteins are abundant in the bacterial cell, and are positively charged, which improves their detection by MALDI-TOF MS, and makes the identification more efficient.

Recent studies show how this new technology offers extraordinary reliability and speed in the identification of different types of microorganisms, including the most frequent human pathogens (staphylococci, streptococci, enterobacteria, etc.), but also infrequently isolated microorganisms whose identification may be difficult and time consuming by conventional methodology [6].

The time required for identification, which using conventional methodology can be at least 24–48 h, compared with just 1 h using MALDI-TOF MS, is a critical factor in clinical diagnosis, especially in severe infections, in which rapid identification of the microorganism is an essential factor in establishing an empirical treatment with a greater probability of success. A study published by our group showed correlation levels of this methodology, in comparison with classical identification methods, in bacterial identification from colonies, of 100%, at the species level, in Gram-positive, and 87.8% at the species level, and 98.8% at the genus level, in Gram-negative [7].

Interest in this method acquired even greater importance when it was observed that identification with a similar level of reliability could be achieved directly from clinical samples, at least in some types of samples such as urine, or directly from blood cultures. Different studies [8], including some carried out by our group [9,10] showed good results in urine cultures with high bacterial loads, and showed that, in more than 90% of cases, a reliable identification could be obtained from positive blood cultures in less than 1 h, once they were detected as positive. In fact, direct identification based on a positive blood culture has already become a common practice, which opens the possibility of establishing much better targeted treatments from the moment the blood culture is positive, and of de-escalating empirical treatments much earlier.

However, the possibilities of this methodology are probably only beginning to be exploited. The study of the databases allows us to observe how microorganisms belonging to the same genus and species show considerably different protein profiles.

A publication in *Future Microbiology* already suggests the possibility of using this type of proteomic technique to establish new, more reliable classifications (molecular serotypes) in *Streptococcus pneumoniae* [11].

MALDI-TOF MS has quickly established itself as a reference technique for the identification of bacteria, including bacteria with particular characteristics, such as mycobacteria, and fungi. Commercial databases now include virtually all the bacteria usually found as human pathogens. Nevertheless, its application to the identification of other pathogens such as protozoa, parasites, and to the identification of arthropod vectors, has evolved much more slowly, especially in the area of human parasites and arthropod vectors.

Ticks are blood-sucking arthropods with a worldwide distribution that behave as efficient vectors of a wide group of human pathogens, including bacteria, protozoa, viruses, and even helminths [12,13,14,15,16]. They are capable of parasitizing numerous species of mammals, birds and reptiles. They constitute the second group of vectors of human diseases, after mosquitoes [14,17]. Currently, around 900 species of ticks are known worldwide [18], adapted to the most diverse environments although, in general, ticks are more numerous in environments in which the animals that serve them as hosts abound, which includes both wild and peridomestic and domestic environments.

Parasites that cause human diseases fall into three main categories: protozoa, helminths, and arthropods [19].

Protozoa are unicellular eukaryotes, and cause such prevalent human diseases as malaria, leishmaniasis, amebiasis, giardiasis, Chagas disease, etc. [20].

Helminths are multicellular organisms. Most are digestive parasites of humans and animals, although they can parasitize other organs of the body, or circumstantially invade them as part of their life cycle [20]. Helminth infections caused by nematodes (*Ascaris lumbricoides*, hookworm, *Strongyloides stercoralis*, etc.), cestodes (*Taenia* spp.) and trematodes (*Fasciola* spp. and *Schistosoma* spp.) account for a considerable global burden of disease, and are among the most common infections in the tropics and subtropics.

The diagnosis of these parasites is conventionally performed by microscopic examination of different samples of the host (stool, blood, urine, tissues, etc.). Sample preparation for microscopic examination is laborious and time-consuming, and diagnosis requires trained and experienced personnel. When direct diagnosis is not possible, due to the location of the parasite, as happens, for example, in neurocysticercosis, or because the elimination of parasites, eggs or cysts occurs in small amounts and irregularly over time, serological diagnosis may be a good alternative [21]. Parasitosis such as fasciolosis, hydatid disease or toxoplasmosis are usually diagnosed using serology.

More recently, molecular techniques (PCR, LAMP, etc.) have been gaining ground due to their sensitivity and specificity, becoming increasingly important in parasitological diagnosis [22].

Finally, MALDI-TOF MS has also shown a potential usefulness for the identification of parasites. MALDI-TOF MS has been applied in recent years, with good results, to the identification of protozoa, helminths and arthropods with good results, although its penetration in the clinical diagnosis of human parasites is still scarce, probably due to the lack of commercial databases that can act on them with the same ease and speed as with the diagnosis of bacterial and fungal infections [23,24].

## 2. Protozoa

### 2.1. Leishmaniasis

Leishmaniasis is a zoonosis caused by obligate intracellular parasites belonging to *Trypanosomatida*, a group of unicellular organisms with modified mitochondrial organelles and a single flagellum. Leishmaniasis is transmitted to humans by bites from an infected female sand fly. Depending on the geographic area, different species can infect humans, producing a variety of diseases.

In endemic areas, the diagnosis may be performed on clinical grounds. Visceral leishmaniasis usually presents with fever, weight loss, anemia, leukopenia, hypergammaglobulinemia, hepatomegaly and splenomegaly. Cutaneous leishmaniasis presents with a history of exposure in an endemic area associated with one or more chronic skin lesions. No vaccine is available for leishmaniasis, so early identification of the infecting *Leishmania* species, and early initiation of antimicrobial treatment, is the only way to control the disease [25].

In most endemic areas of the world, laboratory testing (microscopy, culture, PCR, antigen tests, and serology) is very difficult to obtain. Though microscopy, culture in NNN medium and serological tests remain to be used in many centres, molecular methods are more specific and sensitive than these classical methods, especially in mucocutaneous leishmaniasis [26,27,28,29,30,31]. Molecular methods based on real-time PCR, RFLP, MLST, and multiple genes sequencing [32,33,34] are the reference methods in centers that have the necessary technology to use them [35].

Some recent studies have used MALDI-TOF MS for the identification of different *Leishmania* species from in vitro cultures [36,37,38]. A specific sample preparation method has been described [38] for the identification of various species of *Leishmania* by MALDI-TOF MS. The method was based on needle aspiration of the lesion, and inoculation of the aspirate in an NNN medium or similar, suitable for the cultivation of *Leishmania*. The medium was periodically observed microscopically and, if amastigotes were observed, the content was washed, resuspended in water/ethanol and centrifuged. From here, the methodology was similar to that used with bacteria and fungi. The pellet obtained was resuspended in formic acid and acetonitrile, centrifuged again, and the supernatant applied to the spectrometer plate together with the matrix (α-cyano-4-hydroxycinnamic acid).

The analysis of the protein patterns allowed both the differentiation of the subgenera *Viannia* or *Leishmania*, as well as identification at the species level, with reliability similar to those of the reference methods [38]. This method has subsequently been corroborated by other authors [37]. Other authors have described a method in which, as has also been shown in studies on bacteria, the extraction step with formic acid and acetonitrile is avoided, directly depositing the amastigotes culture together with the matrix on the reading plate [36]. In both cases, the main drawback was the need to culture the sample for several days before being able to apply the MALDI-TOF MS, which significantly slows down the obtainment of results.

### 2.2. Enteric Protozoa

Enteric protozoa, such as *Giardia* and *Cryptosporidium*, are among the most frequent human parasites, even in developed countries. The diagnosis is usually made by microscopy from filtered and concentrated stool samples, which is a cheap, but laborious, time-consuming method, and dependent on the preparation and experience of the microscopist.

*Giardia lamblia* is probably the most frequently diagnosed enteric protozoa in developed countries. Giardiasis may by asymptomatic, but usually presents as a diarrheal disease characterized by abdominal pain and a moderate number of depositions (3–5/24 h) of foul-smelling, greasy stools, beginning 1–2 weeks after infection.

Though *Giardia* trophozoites can be observed microscopically, especially in duodenal aspirates, the diagnosis is usually conducted by observing cysts in stools by microscopic examination, which cannot predict the species or indicate the viability of the cysts. Some 15 years ago, a research group described a MALDI-TOF MS-based approach for the identification of *G. lamblia* and *G. muris* from intact cysts [39]. As has also been observed for other protozoa, the identification can be readily obtained without specific protein extraction. These authors first washed intact cysts and then suspended them in distilled water. They mixed the cysts suspension with the same volume of matrix solution (3,5-dimethoxy-4-hydroxycinnamic acid), incubated for 60 min, and plated on the MALDI plate. After air-drying the plate, mass spectra were acquired, observing that mass spectra for *G. lamblia* were homogeneous and adequate for identification, and mass spectral fingerprints of *G. lamblia* and *G. muris* were distinct enough to allow their discrimination by MALDI-TOF MS.

As happens with other parasitological diagnoses, the main problem with MALDI-TOF MS is that most of the studies are conducted under experimental conditions, or on filtered and purified cysts, which makes it difficult to apply directly to diagnosis, and puts this method at a clear disadvantage with respect to molecular methods. It would be very useful to know, and is one of the studies that needs to be carried out, if a simple filtering and centrifugation of the patient’s stools, similar to those routinely used for microscopic studies, would allow enough sensitivity and specificity to be applied directly in the clinic with a simple and fast preparation process.

The possibility of using MALDI-TOF MS for the identification of enteric protozoa was first suggested for *Cryptosporidium parvum* in 2000 [40]. *C. parvum* causes diarrhea, especially in children, which frequently occurs in outbreaks, and can cause prolonged and severe diarrhea in immunosuppressed patients. The authors of [40] used intact oocysts, and oocysts subjected to freeze–thaw cycles. The samples were applied to the plate without previous extraction, and mixed directly with the matrix. Identification was more effective in oocysts that had been frozen and thawed. Subsequent studies showed that the protein profiles found for *C. parvum* were homogeneous between different samples or origins, and clearly distinguishable from other *Cryptosporidium* species such as *C. muris* [41].

Some methodological variations have been suggested more recently, which seem to give rise to clearer and more differential protein profiles [42].

MALDI-TOF MS has also been evaluated for identification of other intestinal protozoa of clinical human interest, such as *Blastocystis* [43].

*Blastocystis* spp. are common intestinal parasites of humans and animals, with a worldwide distribution. It is considered a frequent infection in humans, although in most cases it is asymptomatic. [44]. Depending on the geographic location, *Blastocystis* may be detected in up to 40% of fecal specimens. Human-to-human, animal-to-human, and waterborne modes of transmission have been proposed [45,46,47,48].

Its role as a pathogen in humans is still under discussion, and it is possible that there are genotypes, and even species, that are pathogenic and non-pathogenic [49]. Often the criteria used to treat a *Blastocystis* infection is based on the presence of significant numbers of protozoa and the absence of other pathogens. There are several possible diagnostic methods for *Blastocystis*, such as culture or serological diagnosis. However, the most used diagnostic method is microscopic observation of stool, although its sensitivity is lower than that of more recently developed methods, such as molecular methods.

Martiny et al. [43] described two methods of MALDI-TOF MS for the identification and differentiation of *Blastocystis* subtypes. They compared, as has been also published for bacteria identification [50], ethanol/formic acid extraction and direct deposition for *Blastocystis* identification. Both studies used conventional extraction and direct application procedures, such as those used in bacterial identification.

In this comparison, mass spectra of *Blastocystis* isolates obtained after ethanol/formic acid extraction were better than direct deposition method. These results suggest that MALDI-TOF MS may be a valuable tool for *Blastocystis* identification and subtyping [43]. The main problem with these studies, as has already been mentioned with others related to other protozoa, is that they were not based on clinical samples, but rather on concentrates obtained in vitro. The method suggested by the authors of [43] is simple and fast enough to be used at the clinical level, as long as it maintains a sensitivity and specificity similar to the centrifugation/filtration systems that are commonly used to concentrate clinical samples. This is a type of study for which there are hardly any publications, and it would be extraordinarily useful to know the real usefulness in clinical samples.

*Entamoeba histolytica* is among the most common of protozoal infections worldwide. Conventionally, the parasite is detected by microscopy and culture, which detects both pathogenic *Entamoeba histolytica* and nonpathogenic *Entamoeba*. Although there are morphological differences that, in general, allow pathogenic amoebas to be differentiated from non-pathogenic ones, it must be considered that *Entamoeba dispar*, a non-pathogenic species, is morphologically identical to *E. histolytica* [51]. As has been described with other protozoa, the emergence of molecular techniques derived from PCR has greatly improved sensitivity, specificity, and the capacity to differentiate species. [52].

Recently MALDI-TOF MS has also been applied to describe the applicability of identification and differentiation of *E. histolytica* and *E. dispar* grown in cultures from clinical samples [53]. The cultures were quantified to 10^6^ trophozoites/mL, proteins were extracted with conventional methods (ethanol/formic acid extraction procedure), spotted on the MALDI plate, and mixed with the matrix. *E. histolytica* and *E. dispar* mass spectra showed several peaks (at least five) apparently able to discriminate, at least, these two species. According to the comments expressed by the authors, the identification of *Entamoeba* species was significantly cheaper by MALDI-TOF MS, compared with the use of molecular techniques, but, on the contrary, the sensitivity was much higher in the case of the latter. An essential question that remains unresolved in this study is the extent to which this greater sensitivity is relevant from the clinical point of view, which would essentially depend on the mean volume of cysts present in a conventional clinical sample, after the use of any conventional filtration-concentration methods used in the clinic [53].

## 3. Arthropods

### 3.1. Fleas

Fleas are small (<5 mm in length), wingless insects, with hind legs modified to allow their characteristic jumping movements. There are some 2000 flea species with varying levels of host specificity, but only a small number have clinical importance. These include the human flea (*Pulex irritans*), the dog and cat fleas (*Ctenocephalides canis* and *Ctenocephalides felis*), the Oriental rat flea (*Xenopsylla cheopis*), and the sticktight flea (*Echidnophaga gallinacea*). They are vectors for several human pathogens such as *Yersinia pestis*, some species of *Rickettsia* and *Bartonella* [54].

Fleas are identified at the species level by morphological examination. This identification, as happens with parasites, requires expertise and is time consuming. Sequencing of the 18S rRNA gene is available and may allow identification, but this technology is expensive and not available in numerous areas.

Yssouf et al. [55] compared the usefulness of different parts of fleas (legs, heads, body, etc.) for rapid MALDI-TOF MS identification of different species of fleas. The study was performed with protein extraction, homogenizing samples in a suspension of formic acid and acetonitrile, centrifuging and mixing the supernatant with the matrix before spotting it on the MALDI plate. The most homogeneous and reproducible protein profiles were obtained from flea body without abdomen, thus, a protein profiles database was elaborated on the basis of these profiles. Samples were then compared against this library, confirming a rapid and reliable discrimination of flea species [55].

More recently there have been some attempts to standardize the processing methodology, both for ticks and fleas [56]. The authors automated the homogenization of the sample, and different conservation conditions for a period of up to six months. Using this method, excellent profiles of fresh specimens were obtained, and it was concluded that the best preservation method was freezing. However, conservation in ethanol, although it gives poorer profiles, is not usually a critical problem for identification.

This possibility of using specimens preserved in ethanol without significant repercussions on the quality of identification has subsequently been endorsed by other authors [57].

Even though the identification of flea species is not a common need in clinical parasitology, since there are databases that apparently offer good results and proven and relatively simple extraction methods, it would be very useful if these databases were commercially available to be added to existing commercial bacteriology, mycobacteriology and mycology databases, thereby expanding the capabilities of MALDI-TOF for identifying clinically important organisms with similar processing procedures.

### 3.2. Ticks

Ticks are obligate parasites, and are potential vectors for several bacterial, viral, and even protozoan pathogens [58,59,60]. Ticks can live in different environments, although they are more abundant in areas where wild animals live or on livestock farms, where the right conditions for the development of their life cycle exist. Animals act as amplifiers and occasionally as reservoirs of some infections. Ticks are usually found in the grass or bushes waiting to feed. Although most tick species are active in the warm months, from spring to fall, some ticks are also active during the winter months.

The spectrum of tick-borne diseases (TBDs) has increased considerably in recent years due to increased clinical and epidemiological surveillance. In addition, the improvement in diagnostic techniques, both in culture and in molecular biology, has contributed significantly. Hard ticks have become the main vectors of infectious diseases in the industrialized world, and the second worldwide. In Spain, different TBDs have been described that can cause systemic manifestations such as Mediterranean spotted fever (caused by different species of *Rickettsia* from the spotted fever group), TIBOLA/DEBONEL (tick-borne lymphadenopathy/dermacentor-borne, necrosis, erythema, lymphadenopathy) caused by *Rickettsia slovaca, Rickettsia rioja* and *Rickettsia raoultii*, and cases of Lyme disease caused by *Borrelia burgdorferi* s.l. Other less frequent TBDs in Spain are tularemia (*Francisella tularensis)*, anaplasmosis (*Anaplasma phagocytophilum*) and babesiosis (*Babesia divergens* and *Babesia microtii*). In addition, since 2010, the Crimean–Congo hemorrhagic fever (CCHF) virus has been detected in Spain in animal ticks, with the first fatal case in humans being described in the summer of 2016 in the province of Ávila. Currently, studies carried out by our group show the presence of this hemorrhagic virus in the Community of Castilla y León not only in animals, but also in people with no memory of having had a compatible clinical picture, and in patients who are admitted to the emergency room with a febrile syndrome [61,62,63].

Since specific tick species are vectors for certain microbial pathogens, the early identification of ticks extracted from human individuals may give an early clue to the disease potentially transmitted, and orientate post-exposure therapy.

Tick diagnostic methods have not undergone many changes in recent years, and morphological analysis is still the gold standard today. Nevertheless, even for experienced entomologists, damaged or immature specimens can be very difficult to identify morphologically [58].

In recent years, new diagnostic methods such as PCR have been introduced, used above all in research centres [64].

About 10 years ago some studies began to suggest the possible use of MALDI-TOF mass spectrometry for the identification of different species of ticks [65,66,67,68]. The first methods described used technically complex and tedious procedures, not very suitable for clinical diagnosis. Karger in [65] used both adult ticks and nymphs homogenized separately in plastic pestles. The specimens were treated with guanidinium chloride solution, sonicated in a water bath and centrifuged. Then, the extract was acidified with trifluoroacidic acid, concentrated, spotted directly on the MALDI plate and, once dried, overlaid with matrix solution, allowed to dry again, and was read.

Once a reference database was constructed, this method allowed a correct identification of ticks to the species level from adult ticks, nymphs or even larvae. Being engorged did not affect correct identification, and almost any small part of the tick, excepting legs, was suitable for identification.

Shortly after, Yssouf [66] developed a method that specifically used legs specimens for identification. They homogenized and extracted the legs with formic acid and acetonitrile solution, and directly overlaid the MALDI plate with the supernatant of this extraction and matrix solution, allowing a satisfactory identification both with ticks obtained from nature and with ticks extracted from animals and patients. According to the authors, the procedure did not take more than an hour of work.

In an additional study, this same author suggested that, by means of MALDI-TOF EM, it would even be possible to differentiate those ticks infected by *Rickettsia* from those that were not [67]. Apparently, the protein profiles of the extracts of legs of ticks infected, and not infected, by *Rickettsia* would offer sufficiently different and characteristic profiles to be able to make this differentiation. A later study by this same group affirmed that the mixture of proteins, obtained by extraction with acetonitrile and formic acid of the hemolymph of the distal part of the legs, would also allow this differentiation between ticks infected and not infected by *Rickettsia* spp. [68].

Rothen et al. [69] studied this problem in Eastern Africa, where several ixodid tick species share habitats and multiple tick infestations in livestock are frequent. They studied the efficacy of MALDI-TOF MS for the identification of ticks frequent in that area, such as *Ambylomma variegatum*, *Hyalomma* sp., *Rhipicephalus. appendiculatus*, *Rhipicephalus decoloratus*, *Rhipicephalus microplus* and *Rhipicephalus evertsi evertsi*. The process used was similar to the others described previously, using legs homogenization with formic acid, centrifugation, and plating supernatant overlaid with matrix for protein profile obtention. This paper confirmed the usefulness of MALDI-TOF MS for reliable identification of species of closely related Afro-tropical ticks. The mass spectra profiles were highly successful in identifying the various tick species. This paper showed MALDI-TOF MS could be a reliable tool in identifying ticks, even when they belong to species which are highly morphologically and genetically similar.

Some partial studies have been carried out in countries such as Mali [70] or Vietnam [71]. In the study developed in Mali [70], 1333 ticks were collected from mammals in three different locations. Morphological identification classified ticks into six species: *Amblyomma variegatum*, *Hyalomma truncatum*, *Hyalomma marginatum rufipes*, *Rhipicephalus (Boophilus) microplus*, *Rhipicephalus evertsi evertsi* and *Rhipicephalus sanguineus sl*. An amount of 471 of these ticks were randomly selected for molecular and proteomic analyses. Proteomic identification was performed using tick legs, after alcohol preservation, drying or directly, followed by extraction of proteins, and mixing with matrix. In total, concordance with morphological and molecular identification was 99.6%. In 13 of the 15 specimens for which there was a discrepancy between visual identification and MALDI-TOF MS, molecular techniques corroborated the identification obtained by MALDI-TOF MS.

Using molecular methods, 76.6% of the specimens tested were positive for *Rickettsia* spp., 37.6% for *Coxiella burnetii*, 20.8% for *Anaplasmataceae*, and 1.1% for *Borrelia* spp. Contrary to what other authors have published, the comparison of MS profiles of 30 uninfected *Amblyoma variegatum* and 12 *Amblyoma variegatum* infected by *Rickettsia africae*, and the comparison between 36 uninfected *Hyalomma truncatum* and 23 *Hyalomma truncatum* infected by *C*. *burnetii,* showed no differences. The authors suggested that the storage mode—fresh versus alcohol—might play a role in the failure to differentiate between infected and uninfected ticks.

In the study conducted in Vietnam [71], the legs of 361 ticks collected in Vietnam, including 251 Rhiphicephalus sanguineus s.l, 99 Rhipicephalus (Boophilus) microplus, 2 Amblyomma varanensis, 7 Dermacentor auratus, 1 Dermacentor compactus and 1 Amblyomma sp. were analyzed using MALDI-TOF MS. The correlation obtained between the visual identification and the identification by MALDI-TOF MS, was 97%. All the specimens were studied by qPCR for infection with Anaplasmataceae, Piroplasmida, *Borrelia* spp., *Bartonella* spp., Coxiella burnetii, and *Rickettsia* spp. A total of 10.8% of the specimens studied were found to be infected by at least one of these microorganisms. Surprisingly, the authors did not report any data concerning the correlation, or not, of these infections with MALDI-TOF MS-specific modification in the spectrum of these specimens.

## 4. Mosquitoes and Flies

The *Culicidae* (mosquitoes) are distributed worldwide. They contain more than 100 genera and more than 3500 different species, of which only a small part affect humans, and an even smaller part being capable of transmitting diseases. However, some of these diseases are of great importance due to their spread and potential severity (yellow fever, malaria, dengue, chikungunya, zika, etc.), having caused some of the most important epidemic outbreaks in recent years.

The expansion of the habitat of different mosquitoes represents a significant risk from an epidemiological point of view, as they are organisms capable of transmitting numerous diseases, both animal and human. On the other hand, this expansion supposes the appearance of different species of mosquitoes in areas in which they are not habitual, and so their identification can be more problematic due to the lack of experience.

In Europe, the Asian tiger mosquito *Aedes albopictus* and the Asian mosquito *Aedes japonicus* are invading Southern and Central Europe. *A. albopictus* has undergone a dramatic global expansion facilitated by human activities, and now invades many countries around the world. Moreover, the evolution of temperatures and climate around the world suggest that *A. albopictus* will continue to spread beyond its current geographical boundaries [72,73,74].

*A. albopictus* can transmit dengue virus, chikungunya virus, zika virus and filaria worms, and experimentally has been shown to be a competent vector of a number of arboviruses including yellow fever virus, Rift Valley fever virus, Japanese encephalitis virus, West Nile virus, and almost 20 other arboviruses [75,76].

The yellow fever mosquito *Aedes aegypti* has established populations in Madeira and the Black Sea, while other Asian and American mosquitoes such as *Aedes atropalpus* or *Aedes koreicus* are being described locally [77].

Similarly, *A. atropalpus*, a north American invasive mosquito, has been described in Italy [78] and the Netherlands [79]. West Nile virus has been isolated from field-collected *A. atropalpus* [80], and laboratory studies have shown that *A. atropalpus* might be a competent vector not only for West Nile virus, but also for La Crosse virus, Japanese encephalitis virus, Saint Louis encephalitis virus, and Eastern equine encephalitis virus [79].

*A. koreicus*, native of Korea, has been described in Belgium and Italy [81,82]. If we consider its strong overlap with *A. japonicus* both ecologically and genetically, it is probable that this mosquito might survive European winters and spread to other European countries [82].

Mosquitoes, similar to other arthropods, have usually been identified based on their morphological characteristics, through dichotomous keys. In recent years, molecular techniques have emerged as an extremely reliable identification method based, above all, on COI genes, although the method has limitations, especially when dealing with closely related mosquito species [83].

On the other hand, it cannot be forgotten that it is a relatively expensive technology, which does not facilitate its use in this aspect, especially in areas with a low-income level, in which infections transmitted by mosquitoes are more frequent.

The first studies on the application of MALDI-TOF MS were published approximately 10 years ago [84]. These studies, carried out on both laboratory-grown and wild-grown *Anopheles* mosquitoes, demonstrated that MALDI-TOF MS was capable of differentiating *Anopheles* species, even those belonging to the same complex.

As in other studies carried out on different arthropods, the extraction method is simple. The authors of [84] tended not to use the abdomen, due to the interference that it can generate in the protein profiles. In this case, the authors used the head and thorax of mosquitoes, obtaining very similar results. Subsequently, they crushed the parts of the mosquito together with formic acid to achieve protein extraction, to finally mix the extraction supernatant with the usual matrix, deposit it on the plate, let it dry and proceed with its processing.

Other authors have subsequently completed the processing procedures, demonstrating that the legs are also perfectly useful for obtaining identifying protein profiles [85].

However, as it has also been demonstrated in the case of ticks, the use of protein profiles from more than one part of the mosquito’s body, generating databases of independent protein profiles for each, increases the reliability of identification [24].

Other studies have shown that MALDI-TOF MS is also perfectly useful for identifying mosquito species from not only adult mosquitoes [24,56,86,87,88], but also from different larval stages [56,89,90] and even eggs [91] from different *Aedes* species (*A. aegypti*, *A. albopictus*, *A. atropalpus*, *A. cretinus*, *A. geniculatus*, *A. japonicus*, *A. koreicus*, *A. phoeniciae*, and *A. triseriatus*).

### 4.1. Phlebotomus

*Phlebotomus*, or sand flies, are blood-sucking flying insects related to the transmission of different human diseases, such as leishmaniasis, as well as diseases caused by the group of phleboviruses transmitted by mosquitoes, such as the Toscana virus (another group of phleboviruses, the Uukuniemi group, is transmitted by ticks). Phleboviruses are RNA viruses, usually associated in humans with a mild, febrile illness known as ‘three-day fever’ disease. Nevertheless, Toscana virus can cause a severe neurological infection.

*Leishmania infantum*, which causes zoonotic visceral and cutaneous leishmaniasis and phleboviruses, is widespread in the Mediterranean region, while *Leishmania major* and *Leishmania tropica*, which cause cutaneous leishmaniasis, are restricted to Asia Minor, North Africa and the Middle East.

Although the main range of sand flies in and around Europe are the southern and eastern Mediterranean basins, climate change is causing their habitat to expand northwards. On the other hand, population movements are also important. Since the vector exists in southern Europe, it is possible that the entry of migrant populations carrying other *Leishmania* species other than *L. infantum* could lead to the appearance of autochthonous cases due to these species.

*Phlebotomus* are not considered as invasive species. The expansion of their populations is not produced by transport to distant areas by means of transport or commercial shipments, as occurs with other mosquitoes, but by environmental and climatic modifications that widen the areas suitable for their development. This does not mean, however, that its diffusion capacity is less. The evolution of the climate towards less cold winters and less rainfall favors the biology of the mosquito, which has adapted well to peri-urban areas in southern Europe. In fact, the largest outbreak of leishmaniasis in Europe in recent years occurred in Fuenlabrada (Madrid), in a peri-urban area, associated with large colonies of *Phlebotomus* in the vicinity of urban housing blocks [92].

Although, globally, *Phlebotomus perniciosus* is the main transmitter of Leishmania in Europe, more than 10 species capable of behaving as transmitters of leishmaniasis in Europe have been described (*P. alexandri*, *P. ariasi*, *P. balkanicus*, *P. halepensis*, *P. kandelakii*, *P. langeroni*, *P. major*, *P. mascittii*, *P. Perfiliewi*, *P. perniciosus*, and *P. tobbi*) [93]. Given the possible modifications of the predominant species due to ecological and climatic changes, etc., it is of interest to have fast, simple, and reliable methods for their identification.

For the identification of *Phlebotomus* by MALDI-TOF MS, different conservation methods have been used. Some preliminary studies have suggested that conservation in 70% ethanol offered lower quality protein profiles compared with freezing methods [94,95], although other authors have worked with this methodology with acceptable results [94,95,96,97,98]. Some of these studies [36] indicated that, although freezing is probably the ideal method of preserving organisms for study by MALDI-TOF MS, the difficulties that this method entails in field work are greater, with respect to simple preservation in 70% ethanol. Therefore, the fact that preservation in 70% ethanol offers reproducible and satisfactory results can often make it the ideal preservation method, at least in field work.

Some authors have also tried to avoid female sand flies where it is evident that they have recently fed, or to process the abdomen of the insect, due to the possible impact that the presence of blood could have on the protein patterns of MALDI-TOF-MS. In fact, some authors have reported up to 35% identification failures [96] when working with engorged specimens.

However, studies carried out in bacteriology (us) show that, when blood cultures are processed directly, blood does not seem to have a significant impact on the quality of the profiles obtained, so the relevance of using the abdomen or using female sand flies that have recently fed is probably an issue to re-evaluate.

In fact, in these first studies [94], 300 organisms belonging to five species (Phlebotomus papatasi, Phlebotomus (Paraphlebotomus) sergenti, Phlebotomus (Larroussius) perniciosus, Phlebotomus (Larroussius) tobbi, and Phlebotomus (Adlerius) arabicus) were processed. All five Phlebotomus species tested generated reproducible protein spectra with a high number of intense signals within the mass range of 2–25 kDa. The protein profiles obtained were species-specific, with several species-unique peaks that allowed reliable and conclusive species identification and taxonomical classification of the analysed sand flies.

Subsequent studies have confirmed these good results [99]. An amount of 166 field-caught sand fly specimens, morphologically identified as *Phlebotomus perniciosus* (56 specimens; 26 males and 30 females), *Phlebotomus neglectus* (four male specimens), *Phlebotomus sergenti* (six specimens; four males and two females) and *Sergentomyia minuta* (100 specimens; 45 males and 55 females), were studied using MALDI-TOF MS. An amount of 149 specimens (89.8%) allowed the construction of a consistent species-specific protein spectra, generating perfectly useful databases as identification reference for *P. perniciosus* and *S. minuta*. In the cases of *P. neglectus* and *P. sergenti*, these databases could not be built due to low number of specimens.

Similarly, a recent study in Guyana used 206 sandflies specimens [100], belonging to 20 species, to build an initial mass spectral library (MSL), and subsequently a group of 199 specimens, belonging to 24 different species, for its validation. Of all the sand flies tested by MALDI-TOF MS in the validation panel, 79% (157/199) gave interpretable MALDI-TOF MS-based identification results. Of the 42 failures, 37 samples corresponded to species molecularly identified that were missing in the MSL. When a corresponding reference spectrum was available in the MSL, 97% (157/162) of the MALDI-TOF MS-based identification results were interpretable.

Probably one of the biggest current problems for the generalization of the use of this technology for the identification of these arthropods is the non-availability of universally approved mass spectral libraries (MSL), as is the case with the libraries available for bacteriology and mycology by the companies that sell the different mass spectrometry equipment. The existence of standardized and updated databases, either from companies or from healthcare organizations, would be highly desirable, preventing each research group from having to create its own database, since it would greatly reduce the workload and allow much more homogeneous results, both at the level of identification and at the level of epidemiological studies that allow knowing the prevalence of each species in different areas, and the movements of populations.

### 4.2. Culicoides

*Culicoides* are small flying insects that can be vectors of important human infections in some geographical areas, such as Oropouche virus and different heartworms (*Mansonella* spp.). MALDI-TOF MS has also been shown to be an effective method for identifying *Culicoides* species [101,102]. As in the case of other vectors, it has been shown as useful for identifying both the adult and larval stages. MALDI-TOF-MS has been used to identify species that are not vectors for humans, such as *Culicoides nubeculosus* [102,103], suggesting that it could also be useful for vectors of human infections.

### 4.3. Triatomines

Triatomines are hematophagous insects present mainly in the Americas, although there are some species in Asia [104]. The most significant disease transmitted by triatomine bugs is American trypanosomiasis or Chagas disease, caused by *Trypanosoma cruzi*, and present in numerous countries in Central and South America, with a very high prevalence in some areas of the South American “altiplano”. It is estimated that there are currently between 6 and 7 million infected patients [105]. In Latin America, *T. cruzi* is transmitted primarily through contact with infected feces or urine of blood-feeding triatomines. The triatomine bugs that harbor the parasites live in the cracks and voids of walls and roofs of houses and outdoor structures, in rural and suburban areas. They usually remain hidden during the day and become active at night to feed on the blood of mammals, including humans. They usually bite exposed areas of skin and defecate and/or urinate close to the bite. Parasites enter the body when the bitten person instinctively rubs the bitten area, and contaminates the bite, open skin lesions, eyes or mouth with feces or urine. Less commonly, the parasite can be transmitted through food contaminated with feces or urine of infected triatomine bugs, through blood transfusions or organ donations from infected patients, and during pregnancy, if the mother develops parasitemia during pregnancy. Cases of contagion such as laboratory accidents have also been described. Although it is a disease that was classically almost confined to South America, population movements, fundamentally migratory movements, have led to an increasing number of cases being described, usually chronic, in other areas, mainly North American and European countries [106].

As in the case of other vectors, the identification has been fundamentally morphological, which implies the need for a significant degree of expertise, and supposes a slow and sometimes difficult diagnosis if the specimen shows some degree of deterioration. Therefore, this is an area in which MALDI-TOF MS could also represent a considerable advance. However, there are few studies related to the morphological identification of triatomines. A study carried out in Guyana [107] comparing triatomines obtained from nature, laboratory-reared and dried, showed that all could become useful for identification, providing, obviously, that the protein spectrum was in the reference database. This brings us back to the need to have, as occurs in bacteriology and mycology, reference databases as wide as possible in terms of genera and species, and accessible at a global level, so that it is not necessary for each author to build their own individual reference databases.

Studies carried out in Brazil also demonstrated the ability of MALDI-TOF MS to discriminate species of triatomines [108,109], although in this case, limited to specific species.

### 4.4. Lice

Human lice are ectoparasites capable of developing all their phases associated with the skin and appendages of man. Some are associated with the transmission of human diseases. Thus, the body louse (*Pediculus humanus corporis*) is associated with various diseases, usually linked to very deteriorated socio-sanitary conditions, such as epidemic typhus, caused by *Rickettsia prowazeckii*, or trench fever, caused by *Bartonella quintana*. As in other cases, morphological identification, which is usual, requires a high degree of expertise, is time-consuming and does not always allow reliable identification, especially if the specimen is damaged. A study in Algeria [110] has recently been published with more than 400 specimens belonging to 14 species of lice (*Bovicola bovis*, *B. ovis*, *B. caprae*, *Haematopinus eurysternus*, *Linognathus africanus*, *L. vituli*, *Solenopotes capillatus*, *Menacanthus stramineus*, *Menopon gallinae*, *Chelopistes meleagridis*, *Goniocotes gallinae*, *Goniodes gigas*, *Lipeurus caponis* and laboratory-reared *Pediculus humanus corporis*).

The authors tested two protocols, one of them longitudinally sectioning the louse and the other separating the cephalothorax, including the legs, from the abdomen, and using the fragment containing the cephalothorax and legs for MALDI-TOF MS. In both cases, the parts were subjected to a grinding process with glass beads and extraction with formic acid and acetonitrile, then proceeded to centrifuge the sample and deposit the supernatant, together with the matrix, on the spectrometer plate. Once the MSL was prepared, more than 400 specimens from different species were identified, based on MALDI-TOF MS. In total, the percentage of correct identification using MALDI-TOF MS was greater than 95%, with the sole exception of *Bovicola caprae* (76%). The percentage of correct identification of *Pediculus humanus corporis* ranged between 96.8% and 100%.

## 5. Pathogen-Harbouring Vectors

A particularly interesting possibility is of being able to not only identify the arthropod vector, but also to determine whether it is a carrier of specific pathogens.

It has already been shown, in two studies published in 2015, that there is the possibility of differentiating mass spectra from ticks infected and not infected by different species of *Rickettsia*. In one of these studies [67], the authors infected *Rhipicephalus sanguineus* and *Dermacentor marginatus* with *Rickettsia conori* and *Rickettsia slovaca*, showing that MALDI-TOF MS was not only capable of reliably identifying arthropods, but also that it was possible to establish specific peaks for each of the species infected by the different species of *Rickettsia*.

In other study by the same group [68], hemolymph MS spectra from *R. sanguineus* infected, or not, by *Rickettsia conorii* were compared, to detect changes of protein profiles. Comparison of MALDI-TOF MS spectra profiles from a leg hemolymph protein mixture of non-infected, and *R. conorii*-infected, *R. sanguineus* specimens showed an MS pattern specific to the infection status. The same results were obtained when fresh specimens of *R. sanguineus*, infected and non infected with *R. conorii*, were studied.

A recent study [111] determined the capacity of MALDI-TOF MS to discriminate between *Anopheles* mosquitoes infected and not infected by *Plasmodium*. Malaria is a febrile illness caused by parasites of the genus *Plasmodium*, which are transmitted to humans during the blood meal of an infected female *Anopheles* mosquito. *Plasmodium falciparum* is the most virulent among the five species affecting humans. A fundamental tool for the control of the disease is the control of mosquito populations, and of the rates, within them, of mosquitoes infected by *Plasmodium*.

Classically, the identification of these mosquitoes is morphological, which has the limitations already mentioned for other vector arthropods (expertise, slowness, impossibility of differentiating species belonging to the same complex, etc.). It should be noted that, for example, in the case of the *gambiae* complex, not all species are vectors of *Plasmodium*, so exact identification may be important. Both *Anopheles* (*A. stephensi*) and *Plasmodium* (*P. bergheri*) species were used in this study, which may be a limitation in that it does not represent the species most commonly associated with human malaria. With this caveat, the results were excellent, since the conclusions obtained from the MALDI-TOF MS and the PCR had a correlation of 100% in the uninfected mosquitoes, and 92.8% in the infected ones (overall correlation, 98.7%).

A recently published study [112] experimentally infected cat fleas (*Ctenocephalides felis*) with *Bartonella quintana* and *Bartonella henselae*. In both cases, it was possible to locate peaks that were specific to the presence of one or another species of *Bartonella*, making it possible to effectively differentiate which fleas had been infected by these microorganisms, and which had not.

However, the study recently carried out in Mali and already cited above [70], found no differences between the DM of ticks infected and not infected by different microorganisms. It will probably be necessary to exert effort for homogenization and systematization to establish procedures both for extraction and for obtaining systematized profiles that allow knowing the exact capacity of MALDI-TOF MS to discriminate between infected and non-infected vectors.

## 6. Other Applications

There are alternative applications based on the same technology as MALDI-TOF MS. Matrix assisted laser desorption/ionization mass spectrometry imaging (MALDI-MSI) is a widely used methodology for rapid visualization of biomolecules in tissues.

Recent studies applied MSI to evaluate lipid metabolism in the cutaneous lesion caused by *Leishmania mexicana* infection in susceptible mice, describing altered lipid profiles in quiescent and replicative amastigotes [113]. Altered lipid profiles have also been described, by using the same method, in livers infected by *L. donovani* [114].

Another recently published application [115] was the control of populations of *A. aegypti* infected by *Wolbachia*. *Wolbachia* is an endosymbiotic bacterium that reduces viral replication in *A. aegypti*. A strategy has been developed to reduce the transmission of viral infections by *A. aegypti*, which consists of releasing mosquitoes artificially infected with *Wolbachia* in natural *A. aegypti* populations. Monitoring of *Wolbachia*-infected *A. aegypti* is a crucial requirement for the effectiveness of the program, since *Wolbachia* infections should remain at high frequencies for the program to be effective. MALDI-TOF MS, coupled with machine learning methods, was very effective for the evaluation of infected populations (sensitivity  =  93%, specificity  =  99%), and, therefore, represents a promising method to evaluate the prevalence of *Wolbachia* in field *A. aegypti* mosquitoes.

Overall, MALDI-TOF MS seems to offer very promising results for the identification of numerous protozoa, helminths, and arthropods, with relatively simple extraction procedures and results with a reliability close to that of molecular techniques. Perhaps the main drawback, at this time, is the unavailability of common databases, maintained with standardized procedures, as occurs in the fields of bacteriology and mycology.

In this regard, a study published in 2017 [116] constructed a free, on-line reference mass-spectral library (MSL) for *Leishmania* isolates, accessible through a free Web-based application, including mass-spectral data for 33 Leishmania species. Four laboratories on two continents evaluated the performance of MSL, and all *Leishmania* strains, but one, were correctly identified at least to the complex level. The application was reliable, and identification results were comparable to those obtained with reference methods. While there are no homologated, commercial databases available through the main MALDI-TOF mass spectrometers, this could be a good alternative, to initiate a common methodology and databases, which will allow more homogeneous results.

## 7. MALDI-TOF MS Cost

A problem that can be important, especially in areas where both parasites and diseases transmitted by arthropods are more frequent, is the cost incurred from the acquisition of the equipment and, where appropriate, of the databases. The cost of the equipment is high, but it is also true that, at least in the clinical field, the use of this equipment for the identification of bacteria and fungi is becoming more widespread, so that its use for the identification of parasites and arthropods results in minimal additional cost. On the other hand, at the moment there are no commercial databases, and the availability of homogeneous databases, with free access and with well-established processing protocols, would be highly desirable. However, in non-care settings, MALDI-TOF MS is a technology that is useful in very different settings, so it can be established as a central service shared by very diverse scientific areas, which can make its cost more affordable. Nor should we forget the advantages it provides, in terms of speed of identification, time savings for technical and expert personnel, and reliability of identification in the case of structurally-deteriorated samples. Other techniques that may have similar advantages in terms of speed and reliability, such as molecular techniques, do not involve such a high initial investment, but involve a much higher cost per determination, which in the medium term may exceed, overall, that of initial investment in a mass spectrometer. On the other hand, if the ability of MALDI-TOF MS to reliably identify arthropod vectors infected by different human pathogens is confirmed, this would undoubtedly be a decisive asset for its routine introduction in diagnosis.

## Data Availability

There is no data other than those obtained from the bibliography appearing in the references chapter.
